# Accompanying patients in clinical oncology teams: Reported activities and perceived effects

**DOI:** 10.1111/hex.13710

**Published:** 2023-01-26

**Authors:** Marie‐Pascale Pomey, Jesseca Paquette, Monica Iliescu‐Nelea, Cécile Vialaron, Rim Mourad, Karine Bouchard, Louise Normandin, Marie‐Andrée Côté, Mado Desforges, Pénélope Pomey‐Carpentier, Israël Fortin, Isabelle Ganache, Catherine Régis, Zeev Rosberger, Danielle Charpentier, Lynda Bélanger, Michel Dorval, Djahanchah P. Ghadiri, Mélanie Lavoie‐Tremblay, Antoine Boivin, Jean‐François Pelletier, Nicolas Fernandez, Alain M. Danino, Michèle de Guise

**Affiliations:** ^1^ Research Centre of the University of Montreal Hospital Centre Montréal Quebec CA; ^2^ Centre d'excellence sur le partenariat avec les patients et le public Montréal Quebec CA; ^3^ Department of Health Policy, Management and Evaluation, School of Public Health University of Montréal Montréal Quebec Canada; ^4^ Centre Hospitalier Universitaire‐CHU de Québec‐Université Laval Québec Québec Canada; ^5^ Centre Intégré Universitaire de santé et services sociaux de l'Est‐de‐l'Île‐de Montréal Hôpital de Maisonneuve‐Rosemont Montréal Québec Canada; ^6^ Institut national d'excellence en santé et services sociaux (INESSS) Montréal Québec Canada; ^7^ Université de Montréal—Faculté de Droit Montréal Québec Canada; ^8^ Gerald Bronfman Department of Oncology, Lady Davis Institute for Medical Research Jewish General Hospital & McGill University Montréal Québec Canada; ^9^ Centre Hospitalier Universitaire de Montréal (CHUM) Montréal Québec Canada; ^10^ Université Laval—Faculté de pharmacie Québec Québec Canada; ^11^ Centre de recherche du CHU de Québec‐Université Laval Québec Québec Canada; ^12^ Centre de recherche du CISSS Chaudière Appalaches Lévis Québec Canada; ^13^ Department of management HEC Montréal Montréal Québec Canada; ^14^ Faculté des sciences infirmières Université de Montréal Montréal Québec Canada; ^15^ Institut universitaire en santé mentale de Montréal Montréal Québec Canada; ^16^ Department of Family and Emergency Medicine, Faculté de Médecine Université de Montréal Montréal Québec Canada; ^17^ Centre intégré de santé et de services sociaux de la Montérégie‐Ouest St‐Hubert Québec Canada; ^18^ Yale Program for Recovery & Community Health New Haven Connecticut USA

**Keywords:** accompanying patients, clinical team, oncology, patient advisor, patient care experience, peer support

## Abstract

**Introduction:**

Since 2018, four establishments in Quebec, Canada, have decided to implement the PAROLE‐Onco programme, which introduced accompanying patients (APs) in healthcare teams to improve the experience of cancer patients. APs are patient advisors who have had a cancer treatment experience and who conduct consultations to complement the service offered by providing emotional, informational and educational support to patients undergoing treatments (e.g., radiotherapy, chemotherapy, surgery), mostly for breast cancer. We aimed to explore the evolution of APs' perspectives regarding their activities within the clinical oncology teams as well as the perceived effects of their intervention with patients, the clinical team and themselves.

**Methods:**

A qualitative study based on semistructured interviews and focus groups was conducted with APs at the beginning of their intervention (T1) and 2 years afterwards (T2). The themes discussed were APs' activities and the perceived effects of their interventions on themselves, on the patients and on the clinical team.

**Results:**

In total, 20 APs were interviewed. In T2, APs' activities shifted from listening and sharing experiences to empowering patients by helping them become partners in their care and felt generally more integrated into the clinical team. APs help patients feel understood and supported, alleviate stress and become partners in the care they receive. They also alleviate the clinical team's workload by offering a complementary service through emotional support, which, according to them, helps patients feel calmer and more prepared for their appointments with healthcare professionals. They communicate additional information about their patients' health journey, which makes the appointment more efficient for healthcare professionals. When APs accompany patients, they feel as if they can make a difference in patients' lives. Their activities are perceived by some as an opportunity to give back but also as a way of giving meaning to their own experience, in turn serving as a learning experience.

**Conclusion:**

By mobilizing their experiential knowledge, APs provide emotional, informational, cognitive and navigational support, which allows patients to be more empowered in their care and which complements professionals' scientific knowledge, thereby helping to refine their sensitivity to the patients' experiences.

**Patient or Public Contribution:**

Two patient–researchers have contributed to the study design, the conduct of the study, the data analysis and interpretation, as well as in the preparation and writing of this manuscript.

## 1 INTRODUCTION

It is estimated that in 2022, 60,000 Quebecers were diagnosed with cancer, which represents 158 new cases per day.^1^ This number has been on the rise for several years and is expected to continue rising in the coming years due to testing delays and backlogs following the pandemic.^2^ Cancer is the country's leading cause of death, and Quebec is one of the provinces in Canada with the highest incidence and prevalence of cancer.^3^, ^4^ In this context, cancer prevention and treatment are a public health priority. In response, Quebec has a cancer directorate within the Ministry of Health and Social Services that has adopted multiple measures to reduce the incidence and prevalence of cancer, but also to improve the quality, safety and experience of care and services. The care and service partnership^5^ constitutes one way of achieving these goals by recognizing patients' experiential knowledge, status as full members of the care team and capacity for self‐determination to make decisions about themselves based on their needs and values.^6^

Moreover, the assessment of cancer patients' experience highlighted that emotional support was the most lacking aspect among the six areas of patient experience assessed in health and social service organizations in Quebec and across Canada.^7^ This need is all the more significant in the context of a pandemic where patients expect and hope to receive emotional support and benevolent accompaniment. In oncology, peer support has usually been provided by ‘patient navigators’ comprised of nurses, social workers, educators, as well as former patients.^8^ By helping patients access healthcare, patient navigators have facilitated and hence accelerated diagnosis and treatment journeys. Patients have benefited from these programmes as it was reported they participated in improving their health by, for instance, increasing adherence to treatment, bringing comfort and guiding them through the healthcare system.^9^, ^10^ This could be considered patient‐centred care, where patients' needs and preferences are integrated into the delivery of care, moving away from medical paternalism.^11^

However, the care and service partnership goes beyond patient‐centered care and can also be exercised at the clinical level by introducing accompanying patients (APs) into the clinical teams to meet patients' need for emotional support.^5^ APs are patient advisors who have acquired specific experiential knowledge related to living with cancer, using services and interacting with healthcare professionals.

They are, therefore, in an optimal position to provide a distinct and unique touch to new patients' support by helping them, for instance, navigate, understand and eventually accept their health situation. APs can also accompany patients to facilitate their transition from acute care to front‐line teams and community cancer teams.^12^ They can improve patients' quality of life by promoting healthy lifestyle habits and reducing symptoms of anxiety and depression^13^ and have positive impacts on healthcare professionals (e.g., work satisfaction, empathy), managers, and decision‐makers (e.g., to better take into account the patients' experience) and the APs themselves (e.g., finalize their recovery).^14^

The PAROLE‐Onco programme aimed to integrate APs into the clinical teams of four different healthcare establishments in Quebec, Canada.^15^ Selected APs were trained and coached to intervene with patients,^15^ while giving them space to innovate in their own ways to accompany patients based on their experiential knowledge. Since 2019, healthcare professionals have introduced, during medical appointments with patients, APs accompanying services as an additional resource, and patients were free to accept or refuse such a resource. Research coordinators or clinical staff members monitored all procedures and collected essential clinical data on patients who had consented to participate in an anonymous and confidential manner to match them with an AP with a similar profile. Patients then made appointments with their AP according to their needs.

To date, the perspective of APs directly involved at a clinical level has been poorly documented. We aim to assess the evolution of APs' perspectives regarding their activities over time when APs and the perceived effects of their intervention on themselves, on the patients and on the clinical team.

## 2 METHODS

Data were collected on two separate occasions, at the beginning of the PAROLE‐Onco programme, where APs started APs (T1), and 2 years later (T2).

### 2.1 Settings

Table 1 presents the four establishments that were included in this study: the Centre hospitalier de l'Université de Montréal (E1), the Centre Hospitalier Universitaire de Québec‐Université Laval (E2), the Centre intégré universitaire de santé et de services sociaux (CIUSSS) de l'Est‐de‐l'Île‐de‐Montréal (E3) and the CIUSSS de la Mauricie‐et‐du‐Centre‐du‐Québec (E4). Each establishment recruited its own APs (29 in total), and one site (E3) set up monthly meetings including a doctor and a psychologist to better accompany APs. Some APs did not have the opportunity to accompany patients since they were involved in the preparation phase before the intervention began. Therefore, they were not included in the data collection. The programmes in which APs were implemented include two in breast cancer (E1 and E4), one in breast oncogenetics (E2) and one in breast and gynaecologic cancers (E3).

**Table 1:** Establishments' characteristics

Establishment	E1	E2	E3	E4
Programme	Breast cancer	Breast oncogenetics	Breast and gynaecologic cancer	Breast cancer
Number of APs in total in T1	5	4	5	3
Number of APs in total in T2	9	2	14	1

Abbreviation: AP, accompanying patient.

### 2.2 Data collection

Data were collected via semistructured interviews and focus group discussions. All APs from the four establishments were invited to participate in T1 and T2. Participants were contacted by telephone or email to participate and to sign electronically the consent form approved by the Research Ethics Committee. No compensation was offered. All participants consented to partake in the research and be recorded. Due to the context of the COVID‐19 pandemic, the interviews were conducted either by telephone or videoconference, and the focus group discussions were carried out by videoconference. The questions in T1 (Supporting Information) aimed to identify, among other information, the roles of APs and the effects of their interventions and were co‐created and pilot‐tested with two patient–researchers (patients included in the research team; M.‐A. C. and M. D.). T1 data collection events were realized 4 months after APs were first introduced in the four establishments. Two years later (T2), the data collection aimed to assess the change in the APs' perspective regarding their roles and the effects of their interventions by presenting the T1 results. APs discussed how elements have changed since the new APs joined the team or whether new elements have emerged. Therefore, no interview guide was used in T2. Transcripts of the interviews and focus group discussions were prepared. All data collection events were carried out in French and were subsequently translated into English.

### 2.3 Participants

In total, for the two rounds of data collection (T1/T2), we were able to interview 20 different APs (T1: *n* = 10, T2: *n* = 10). A summary of data collection in T1 and T2 is presented in Table 2, and Table 3 presents a description of the participants.

**Table 2:** Summary of data collection events

Data collection events	T1	*N* (participations)	T2	*N* (participations)
Focus groups	2	7	5	19
Interviews	8	8	0	0
Total of participations		15		19
Total of participants		10*		16**

Abbreviation: AP, accompanying patient.

* Out of a total of 15 participations, 5 APs participated in 2 events. Therefore, there are 10 different APs in T1.

** Out of a total of 19 participations, 3 APs participated in 2 events. Therefore, there are 16 different APs in T2, of which 6 also participated in T1. Therefore, there were 10 new APs that were interviewed since T1.

**Table 3:** Description of participants

Characteristics		T1	T2
		*N* (participants)	*N* (participants)
	E1	4	7
	E2	1	2
	E3	2	6
	E4	3	1
	25–34 years old	0	1
	35–44 years old	0	2
	45–54 years old	2	2
	55–64 years old	5	7
	65–74 years old	3	4
	Born in the province of Quebec	9	14
	Born outside of Canada	1	2
	Partial studies (high school, college or university)	1	3
	Has a university degree	9	13
	Works part‐time	1	1
	Works full‐time	0	3
	On leave (illness or maternity)	0	3
	Volunteering	2	3
	Retired	7	6
	Breast cancer	7	9
	Oncogenetic trajectory	2	3
	Breast cancer and oncogenetic trajectory	0	1
	Gynaecologic cancers	0	1
	Metastatic cancer	1	2

Abbreviation: AP, accompanying patient.

In T1, of the 10 APs that were involved in the four establishments and that have accompanied patients, all of them agreed to participate. One focus group with E4 was held in June 2019 (*n* = 3 participants). Another focus group was held in September 2019 E1, E2 and E3 (*n* = 4). The two focus groups were led by the principal researcher (M.‐P. P.) and lasted 58 and 178 min, respectively. Moreover, eight individual interviews were held between April and May 2020 and were conducted by research coordinators (K. B. and M. I.‐N.). They lasted between 30 and 63 min. Out of the 10 APs, 5 participated in two data collection events (individual interviews and focus groups).

In T2, of the 20 APs that were APs, 16 agreed to participate (4 did not reply to our invitation). Of the 16 participants, 6 have participated in T1. The other four APs that participated in T1 were not reinvited in T2 because they were no longer involved in the PAROLE‐Onco programme due to personal issues. Therefore, there were 10 new APs that were interviewed in T2. An initial focus group with E1 and E3 (*n* = 3 participants) was held in September 2021 and lasted 35 min. At that time, the APs had been APs for 12–22 months. Four other focus groups for each establishment (*n* = 16 participants in total) were held between March and May 2022 and lasted between 80 and 115 min. The range of months of involvement was between 6 and 32 during this period. The events were led by the principal researcher or a research assistant (J. P.). Of the 16 APs, 3 participated in two data collection events.

### 2.4 Data analysis

To analyse data, we followed the six‐step guideline of Braun and Clarke.^16^ First, all interviews were transcribed to familiarize ourselves with the data. Second, several meetings between the authors, including two patient researchers, took place to construct the codebook that contained four main categories: (1) APs activities regarding patients and clinical teams, (2) PAs perceived effects of their activities on the patients, (3) on the clinical team and (4) on themselves. Then, we used a thematic analysis approach to better ‘understand a set of experiences, thoughts, or behaviors’ pertaining to these categories.^17^ We used an inductive approach to theme identification—or patterned responses that occurred in the data set.^17^ Coding was done using the QDA Miner Software (version 6.0.2.). Steps 4 and 5 consisted of grouping some themes together to define APs activities. The final step is the writing of this manuscript.

## 3 RESULTS

**Figure 1:**
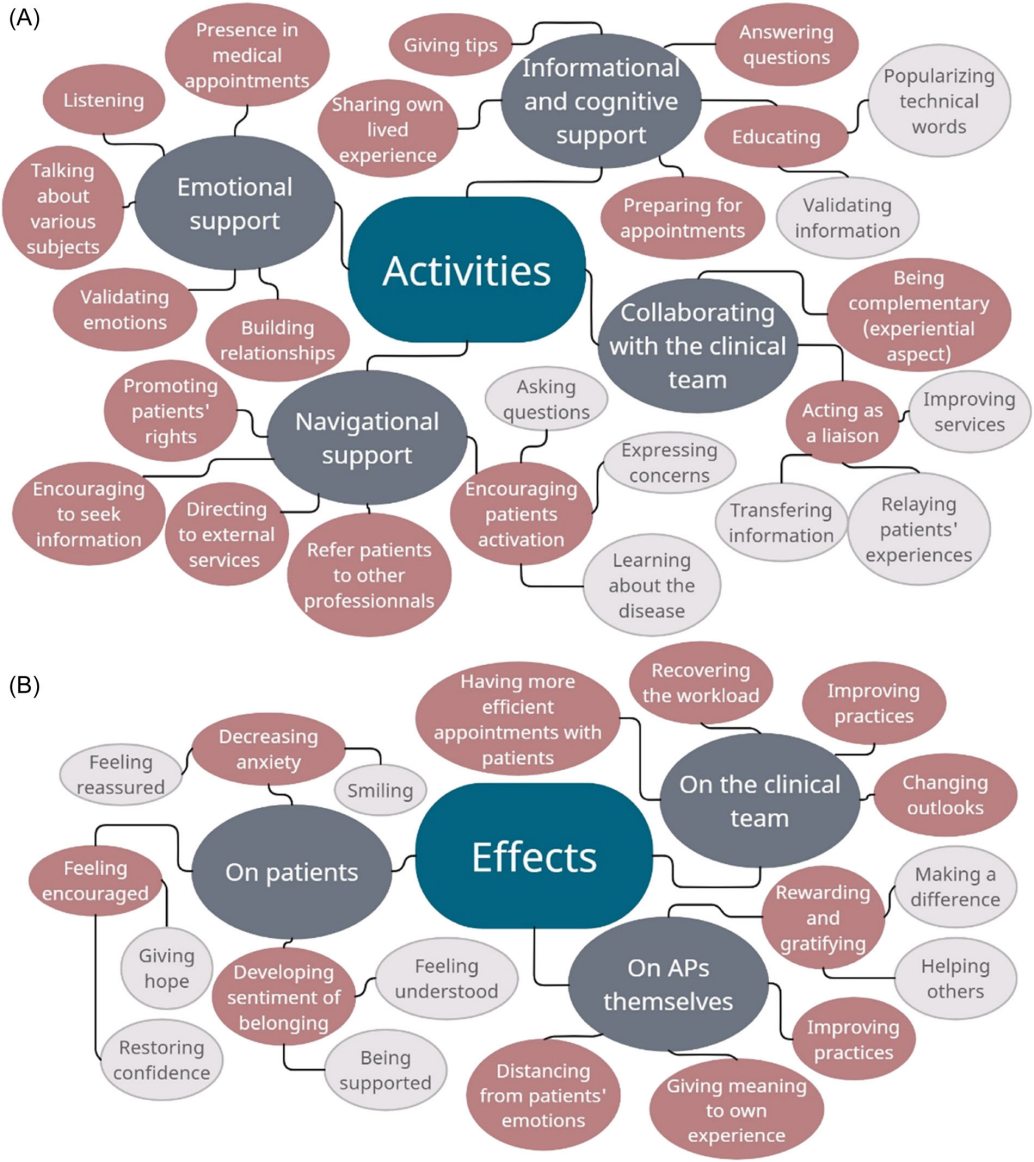
Thematic map

The qualitative analysis enabled us to group APs activities into four categories: emotional support, navigational support, informational and cognitive support and collaborating with the clinical teams (Figure 1A). Elements of responses pertaining to the effects of APs activities can be found in Figure 1B.

### 3.1 Activities

#### 3.1.1 Emotional support

In T1, one of the main roles reported by APs was to listen. Since APs have undergone similar experiences, they can better understand what patients are going through, and therefore lead conversations patients could not have with their loved ones: ‘The fact that we have experienced the situation, we are able to be more empathetic towards patients […]. The patients tell me things that they could not say to a partner because they do not want to disturb them’ (E4‐19). In T2, APs reported moving from an unconditional listening role to a more ‘active’ role towards patients. APs now put the emphasis on discussing and validating the patients' emotions to help them understand and accept their journey with cancer while also reducing their anxiety and reassuring them: ‘what I have experienced with several women is validation, validating them in what they feel, in the choices they can make’ (E2‐01).

Overall, APs try to not only talk about the disease, treatment and care trajectories, but also about the difficulties experienced at home, in interpersonal relationships and in daily activities, thus participating with the patients in building relationships based on trust and openness. Some mentioned in T2 that they have accompanied the patients' loved ones to bring comfort to the whole family. Some also said they have accompanied patients to their medical appointments, especially patients who may have barriers that limit their ability to interact with their physician.

#### 3.1.2 Navigational support

Various resources inside and outside the hospital are offered to the patients, and one of the roles of APs, as mentioned both in T1 in T2, is to act as patient navigators. Not only are they familiar with the range of hospital services offered, but they also ‘know the entire chain of operations for having gone through it’ (E2‐01). APs noticed that ‘often women are not told about this. […] They don't know they have access to this, and they always think that you have to pay too’ (E1‐02). APs therefore ‘encourage them to get the right information’ (E2‐02) and make sure to direct patients to the external services made available to them to complete the support sessions they offer, if needed. They also suggest referring patients to other professionals, be it a psychologist, a nutritionist or a social worker, if they feel that the patients' degree of distress lies beyond their area of expertise and experiences undergone to effectively meet the latter's various needs.

In T2, APs realized that the patients are often not informed of their rights. This touches on building patients' ability to advocate for their own rights. They mention their new role in ensuring they know their rights and become comfortable using them. Encouraging them to ask questions and to assume responsibility for fulfilling their desire to understand and learn about their disease are some of the aspects discussed during the meetings with their patients. Therefore, APs help patients make their own decisions by encouraging them to think through the situation, ask questions and express their concerns and uncertainties: ‘here are patients who are afraid to ask questions because they don't want to be perceived as annoying patients. You always have to reassure them. We say “no, it's your right.” We have to encourage them’ (E1‐05).

#### 3.1.3 Informational and cognitive support

The accompanying sessions with the patients allow APs, in T1, to share their own lived experiences with discernment without making it an example to be followed. Their role is not to teach, but rather to use their own experience as a way to answer patients' questions. Having experienced the system at hand, APs could serve as resource persons for individuals who are unaware of how to navigate a new healthcare structure: ‘We're able to guide them and encourage them. We are not there to pity them and take care of them. We're really there to support them and say, “Look, I've been there. Here are the steps”’ (E4‐19).

In T2, APs explained that they give tips they have learned throughout their own journey with patients instead of giving advice which, according to them, they are not trained to do, nor do they have the expertise to give opinions that include clinical details: ‘I don't like giving advice because I feel it's not part of my mandate… it's really sharing [experiences]’ (E2‐02). Another activity mentioned in T2 is that of helping patients to understand and validate the information received by the clinical team, and thus help them prepare for their appointment with healthcare professionals. Indeed, as opposed to T1, they can help educate patients by popularizing some technical information transmitted by the healthcare professionals and talking to patients using the same language as them: ‘we have the same words because we have often experienced the same emotions, so we will share the same words that the professional will not share’ (E1‐01). Some APs specify that for medical information, patients instinctively know to direct their questions to healthcare providers. With APs, they prefer to ask questions about the establishment and the care pathway: ‘They will ask more questions about their facility: Did you stay in the hospital long? Was it hard? Did you have any pain? That kind of questions’ (E2‐02). By answering patients' questions based on their experiential knowledge, they ‘help patients become partners in their care [by having] a kind of educational role’ (E1‐07).

#### 3.1.4 Collaborating with the clinical team

In T1, some APs felt they were not integrated into the clinical team, but that ultimately it could bring a value‐added resource to healthcare professionals: ‘it would improve the contact they have with their patients’ (E4‐22). In T2, they specify that they have a complementary role with the clinical team with respect to the emotional and experiential aspects of the disease versus the therapeutic aspect provided by the clinical team. Some mentioned that ‘the professionals, they can't know if they haven't lived it … It's just a fact’ (E1‐01). Therefore, APs form a different relationship with patients than healthcare professionals can, and they complete the range of services offered to the establishment. APs felt that they are (or should be) ‘a link in a chain of all the different professionals, that [they] are part of the group’ (E1‐04), although some feel they have not yet fulfilled that role.

Moreover, APs mentioned in T2 that their role also consists of acting as a liaison between the patients and the clinical team. For example, with patients' consent, they can transfer information to the clinical team. It is done by updating them regularly about their patients' health journey and their patients' personal situation through the provision of medical information about treatment and the disease they may not know: ‘we also serve to update the doctor on important facts that can have an impact on the patients' health’ (E3‐07). They can also relay how the patients experience their care and how the healthcare professionals can improve it. Even if information transmission is not homogeneous across establishments, some APs have developed good communication with team members: ‘the pivot nurse was a good ally. I would call her, leave a message, and she would call me back the same day’ (E3‐07).

### 3.2 Perceived effects of their intervention on patients

Both in T1 and T2, APs have the perception that patients are less stressed at the end of a meeting after they were listened to and were able to be reassured: ‘I am always told that “It makes me feel good to talk to you”’ (E1‐10). There is no need for a full session to have that effect, ‘even 5 minutes with a patient in a corridor, in the elevator, the person is happy, she has really lowered her anxiety level’ (E1‐03). After talking with a patient, APs could sense that they were leaving them with a smile: ‘we ended … I'm not telling you with bursts of laughter but with a smile. I'm sure the patient on the other end of the phone line smiled’ (E4‐22). In T2, APs added the fact that their accompanying sessions help restore their patients' confidence and hope, and develop the patients' feeling of belonging as they feel understood and supported in their life experiences: ‘I find it positive for patients to be with other people who have had cancer from which they have recovered, that there is long‐term healing that exists. I find it encourages them to continue’ (E3‐15).

Nonetheless, while in T2, the rapid and positive effects of their support on patients are pointed out, some APs mentioned that a few minutes are not sufficient to delve deep into the patients' concerns and questions, and thus have a positive long‐term impact on them. Sometimes, several meetings are necessary before a certain progression in the patients' journey is seen.

### 3.3 Perceived effects of their intervention on the clinical team

Both in T1 and in T2, APs shared that they could facilitate the task of healthcare professionals by preparing patients to meet and feel comfortable with the information they receive from their physicians. They think that it could be easier for healthcare practitioners to have patients that are calm during a medical appointment: ‘If [the] patient is in a good mood, understands and feels safe because she has been spoken to, it is much easier to care for that patient. She will be a lot more open to treatment. I'm sure of that’ (E4‐22).

In addition, since health teams can be understaffed and overwhelmed, APs can ‘recover a little from the overload of work’ (E1‐02). In T2, they put more emphasis on the complementary aspect of their role to the health professional's therapeutic and curative function through their emotional support and their backing in the process of the patients' adaptation and acceptance of the disease: ‘we're a bit of a buffer between the two; we come to sooth a lot of things that the work staff doesn't always have time to sort out or that the patient doesn't dare to say’ (E3‐02). Also, the information shared between APs and patients could make the appointments more efficient for the clinical team by ensuring that the tasks are separated. This way, patients can be directed to other resources that offer services that the healthcare team may not be able to provide: ‘The health professionals, to advise massage therapy … They didn't have cancer, so the process of reconciliation with the body, they don't know it that much’ (E4‐22).

By relaying how the patients experience their care and how the healthcare professionals can improve it, APs mentioned feeling heard, and receiving openness and appreciation from the clinical team. For example, when patients made suggestions to improve how patients are received at the hospital, APs met with the staff and received positive feedback, and ‘they said it changed their whole outlook. As a result, what I understood was that it was to be an integral part of their training’ (E3‐16). In turn, this link that is created with the clinical team encourages the staff members to ask APs more questions, consult with them and ask for their opinion.

### 3.4 Perceived effects of their intervention on themselves

In T1, they mentioned that being an AP is rewarding, and it satisfies their need to help others: ‘I'm retired, but still feel the need to do things for other people. So that satisfies my needs well. And that's something rewarding’ (E2‐01). They feel like they are making a difference, and this benefits them both as their discussions also serve as a learning experience: ‘It's a plus in both directions. When I talk to someone, it makes me feel just as good to see that I have lightened their mood, as I have helped them. She helps me’ (E1‐04). In T2, the APs discussed how the different patients they encounter represent a learning and experiential opportunity for them to improve their caregiving abilities and skills. Also, having the opportunity to share allows APs to give meaning to their own experience, and helping someone gives them a sense of purpose. However, some APs can find it emotionally difficult to listen to patients' distress: ‘For sure sometimes it can be hard for us. […] We may have lived with cancer, yes, but we haven't experienced all the distress that people can experience’ (E4‐22). But overall, APs in T2 are more capable of distancing themselves from their patients' life stories to prevent their emotions from taking over their role as unbiased listeners. They felt as if they have developed ways to help them maintain control over their emotions and lighten the heaviness of listening sessions, whether through the community of practice meetings they organize between APs, which help them to share ideas about the more difficult encounters they might have, or by adopting the neutral attitude discussed above.

In T2, however, not all APs continue to consider their work as gratifying. Some perceive their role only as an opportunity to give back, which does not necessarily bring them anything personally: ‘The word gratifying is not what resonates with me anymore’ (E1‐07).

## 4 DISCUSSION

The objective of this study was to assess the evolution of APs' perspectives regarding their activities when APs, as well as the perceived effects of their intervention on the patients, on the clinical team and on themselves.

### 4.1 Different activities played

Like many studies on peer support interventions for cancer,^9^, ^10^, ^18^, ^19^, ^20^, ^21^ our study shows that the primary activity of APs is to listen to patients and validate their emotions to facilitate their acceptance process of the disease and increase their ability to fight cancer in a positive way. This is done by sharing their own lived experiential knowledge and tips they acquired throughout their own journey with illness. They also share information not only about their experiences with the disease and treatments but also about community resources, a role that is also reflected in the work of Fisher et al.^22^ and Jacobson et al.^23^ They allow patients to visualize the care pathway and thus gain a better understanding of the different steps they will have to go through. In T2, APs' activities shifted from listening and sharing experiences to empowering patients by helping them become partners in their care. It is possible that the ‘listening role’ is a less threatening first step to finding a place within the care team, but time and experience APs have given APs the ability to try and take on a more active role in the clinical team. Other functions, like advocacy support, are potentially more contentious, and it is not surprising that it appears in T2 rather than in T1. Thus, these APs also have a patient navigator role as presented in the literature,^8^, ^9^ and they are all former patients of the establishment and have all been led through the same trajectory. Another capacity emphasized by APs was their ability to help patients better prepare for their medical appointments and better understand their illness, treatments and the consequences of decisions made. Often patients are reluctant to ask professionals to clarify information provided to them or ask questions, or take their place in the decision‐making process. By playing this role, APs can provide a safe space in which to ask questions.^24^ This educational activity is also found in the literature^25^, ^26^, ^27^, ^28^ but places less emphasis on APs playing a counsellor role. In our context, they help patients to explore coping resources in a nonconfrontational way using reflective listening rather than persuasion. Finally, they can talk about professionals and introduce them to patients in reference to their own experience of the patient‐professional relationship. Such a role is rarely reported in the literature outside of mental health.^29^ Therefore, APs provide meta literacy support,^30^ characterized by support on behavioural (patient behaviour), social‐emotional and cognitive levels, and not only at the educational level.

### 4.2 Particularities to be a member of the clinical team

While there are many studies on the contribution of peer support programmes in cancer care,^9^, ^10^, ^18^, ^19^, ^20^, ^21^ there are few reports that address peer mentoring in which APs are integrated into the clinical team, except in the area of mental health.^31^ Our results show that, in T2, some APs felt more integrated into the clinical team and were able to communicate and collaborate with healthcare professionals, although not all establishments have succeeded in fully integrating APs. Introducing APs as full members of the clinical team translates into APs' having access to the relevant medical information on the patients with their consent to better understand the context of their accompaniment. It also means being able to interact with healthcare professionals when they identify situations that require the contribution of professionals and the possibility of leaving a note in the patient's medical file, with the patient's consent, summarizing the main points of the exchanges that may be relevant for the team.

Being former patients of the establishment and thus being highly familiar with the professionals, APs become the ‘transmission agent’ between the professionals and the patients. On the patients' side, they encourage the development of a bond of trust with the professionals. They also embody hope in the team's ability to care for them, as the APs are there to tell them. For healthcare professionals, the feedback on the patients' health journey and personal life allows them to better understand the patients' reality and thus better respond to their needs to help them have a better experience. Also, APs emphasized the distinction of roles within the clinical team, as they did not consider that discussing treatment and clinical details was their responsibility. They were comfortable giving advice based on their own experience and did not seek to provide professional counselling. APs develop complicity with the patient based on a shared experience. This bond can bring to light important clinical situations that would otherwise not have been reported to the clinical team. By becoming a member of the team, they can suggest that other professionals, such as psychologists, would be able to meet patients' different needs. Again, such a role is not very present in the literature available on peer support programmes except in mental health.

### 4.3 AP's perception of the effects of their interventions

Through this research, we were able to show that the APs had perceived a certain number of effects of their accompaniment to the patients. The first effect that stands out is the decrease in anxiety, whether it be at the time of the examination (genetic, biological, radiological, etc.), the announcement of the diagnosis, the choice of treatments and the end of the treatments. Having a safe place to discuss their fears and anxieties and being supported by people who have successfully dealt with them and are still alive allows them to lower their anxiety levels. By being less anxious, patients are then better able to retain the information given to them, be more able to prepare for their appointment and dare to ask questions. Such a change in patients' behaviour allows them to be more involved in their care, to regain power over their health,^17^ and to develop a partnership with their healthcare professionals.^32^, ^33^ APs foster a bond of trust between the clinical team and the patients by sharing their own relational experiences with the team. This lived experience allows patients to identify with and feel more comfortable communicating with their professionals.^34^ As discussed by Fisher et al.,^22^ one of the key features of peer support revolves around encouraging self‐empowerment, as supporters focus on a person‐centred approach. In T2, APs also emphasized restoring patients' confidence through their accompanying sessions. The authors considered supporters' role in helping patients cope with negative emotions and insecurities, just as APs mentioned discussing with patients their fears and worries.

For professionals, as evidenced by the role of APs within the team, they make them more aware of the patients' perspective and experience and may therefore realize that they may have to change their behaviour, in particular by improving their communication abilities. This contributes to improving the quality of care, as highlighted by Gates and Akabas^35^ and to humanizing the care process.

For APs themselves, Brodar et al.^19^ mentioned that peer supporters could become emotionally charged following their encounters with patients as they can be reminded of their own experience with cancer. It was therefore suggested that there should be more support from clinical staff as well as from other peer supporters to create a sense of community which could comfort APs during difficult times and help them give meaning to their own experiences. However, in our study, such a need did not emerge. This can perhaps be explained by APs meeting regularly in a community of practice where they can share their accompaniments and find support from the other peers present. To APs, APs are seen more as a learning opportunity, which helps give meaning to their own journey with their illness while also giving them a sense of accomplishment. Such a result has been mentioned by Solomon^34^; being a peer provider offered the latter personal growth as it increased their confidence in their capabilities to support and their ability to cope with the illness as well as their self‐esteem.

### 4.4 Limitations

The concept of APs as an integral member of a clinical team is quite recent. Our study is exploratory and requires further study over time and quantitative studies to test different models. We also recognize that APs have different perceptions of their integration, and thus the results may not be an exact representation for all APs, nor do all APs practice every activity mentioned above. Through their own experience and with time, they have developed their own way of APs. Therefore, it would be important to further explore the different accompanying profiles of APs in the future. Similarly, the contexts in the four establishments are different and, accordingly, our results cannot be generalized. Moreover, here we have presented APs' perspective of their roles and their effects on themselves, the patients and the clinical team, but it is also important to assess the challenges and facilitators of their integration into the clinical team. Those results are presented in another manuscript in preparation. Future work could assess how the roles of APs and their effects on their loved ones would change if they were paid as opposed to working as volunteers, as is currently the case. In addition, it would be important to assess the patients' as well as the clinical teams' perspectives on APs. Data collection for the two populations is currently underway. Also, of the 29 APs that were included in the clinical teams at the four establishments, 20 participated in the study because some had changed positions or were unable to respond to our request. However, in our data collection process, both in T1 and T2, we felt that we had reached data saturation.

## 5 CONCLUSION

This article assesses the evolution of APs' perception of their role and the effects they can have on people affected by breast cancer, mostly, on healthcare professionals and on themselves. It highlights that APs provide emotional, informational, cognitive and navigational support that allows patients to be more empowered in their care. As they gain experience, APs progressively endorse a broader set of roles within the teams. APs also help patients become partners in their care. They are able to mobilize their experiential knowledge to complement professionals' scientific and experiential knowledge. By integrating them into teams, they can also help professionals more effectively take into account patients' lived experiences in the way they respond to their needs. In this way, they contribute to improving patients' experience of care, but also the professionals' sensitivity to patients' experiences. However, to be able to respond to patients' needs and fit into teams, organizational factors may be more or less favourable. In a second article, we, therefore, propose to focus on the issues identified by APs and examine how healthcare establishments can further facilitate integrating APs into their team.

## AUTHOR CONTRIBUTIONS

Marie‐Pascale Pomey, Monica Iliescu‐Nelea, Cécile Vialaron, Karine Bouchard, Louise Normandin, Marie‐Andrée Côté, Mado Desforges, Israël Fortin, Isabelle Ganache, Catherine Régis, Zeev Rosberger, Danielle Charpentier, Lynda Bélanger, Michel Dorva, Djahanchah P. Ghadiri, Mélanie Lavoie‐Tremblay, Antoine Boivin, Jean‐François Pelletier, Nicolas Fernandez, Alain M. Danino and Michèle de Guise have conceived and designed the project. Marie‐Pascale Pomey, Jesseca Paquette, Monica Iliescu‐Nelea, Cécile Vialaron, Rim Mourad, Karine Bouchard, Louise Normandin, Marie‐Andrée Côté, Monica Iliescu‐Nelea and Pénélope Pomey‐Carpentier have participated in data collection and analysis. All authors have made substantial contributions to this study and have participated in the writing of this paper.

## CONFLICT OF INTEREST

The authors declare no conflict of interest.

## ETHICS STATEMENT

This study received ethical approval from the Research Ethics Committee (17.260) of the Research Centre of the University of Montreal Hospital Centre (CRCHUM).

## Supporting information

Supporting information.

## Data Availability

Data sharing is not applicable to this article as no data sets were generated or analysed during the current study.

## References

[1] Lacoursière A . Registre québécois du cancer: Le Québec est « dans le noir ». *La Presse*. 2022. Accessed September 12, 2022. https://www.lapresse.ca/actualites/sante/2022-06-14/registre-quebecois-du-cancer/le-quebec-est-dans-le-noir.php

[2] Patt D , Gordan L , Diaz M , et al. Impact of COVID‐19 on cancer care: how the pandemic is delaying cancer diagnosis and treatment for American seniors. JCO Clin Cancer Inform. 2020;4:1059‐1071. 10.1200/CCI.20.00134 33253013 PMC7713534

[3] Santéscope . Principales causes de décès. Institut National de Santé Publique du Québec. 2019. Accessed September 7, 2022. https://www.inspq.qc.ca/santescope/syntheses/principales-causes-de-deces

[4] Société canadienne du cancer . Vue d'ensemble des statistiques sur le cancer. Société canadienne du cancer. 2022. Accessed January 10, 2022. https://cancer.ca/fr/research/cancer-statistics/cancer-statistics-at-a-glance

[5] Pomey M , Morin E , Nault C , Clavel N , Beaumont M , D'Amour M . Engager les patients à tous les niveaux de gouvernance: l'exemple du CIUSSS de la Mauricie‐et‐du‐centre‐du Québec. Le Point en Santé et Services Sociaux. 2017;12(4):12‐17.

[6] Karazivan P , Dumez V , Flora L , et al. The patient‐as‐partner approach in health care: a conceptual framework for a necessary transition. Acad Med. 2015;90(4):437‐441. 10.1097/acm.0000000000000603 25607943

[7] Rossy Cancer Network . P2—Outpatient rating of treatment experience. 2016. Accessed September 7, 2022. https://www.mcgill.ca/rcr-rcn/quality-dimension/p2-outpatient-rating-treatment-experience

[8] Paskett ED , Harrop JP , Wells KJ . Patient navigation: an update on the state of the science. CA Cancer J Clin. 2011;61(4):237‐249. 21659419 10.3322/caac.20111 PMC3623288

[9] Baik SH , Gallo LC , Wells KJ . Patient navigation in breast cancer treatment and survivorship: a systematic review. J Clin Oncol. 2016;34(30):3686‐3696. 10.1200/JCO.2016.67.5454 27458298 PMC5065113

[10] Hoey LM , Ieropoli SC , White VM , Jefford M . Systematic review of peer‐support programs for people with cancer. Patient Educ Couns. 2008;70(3):315‐337. 10.1016/j.pec.2007.11.016 18191527

[11] Dumez V , Pomey MP . From medical paternalism to care partnerships: a logical evolution over several decades. In: Pomey MP , Denis JL , Dumez V , eds. Patient Engagement: How Patient‐Provider Partnerships Transform Healthcare Organizations. Organizational Behaviour in Healthcare. Springer International Publishing; 2019:9‐16. 10.1007/978-3-030-14101-1_2

[12] Centre intégré de santé et de services sociaux de la Gaspésie . Un pont vers la maison: la vie après les traitements contre le cancer. Faisons équipe contre le cancer. 2019. Accessed November 21, 2022. https://cancergaspesie.ca/projets/un-pont-vers-la-maison-la-vie-apres-le-cancer/

[13] Efanov JI , Papanastasiou C , Arsenault J , et al. Contribution of patient‐advisors during rehabilitation for replantation of digits improves patient‐reported functional outcomes: a presentation of concept. Hand Surg Rehabil. 2018;37(18):212‐217. 10.1016/j.hansur.2018.04.002 29793756

[14] Pomey MP , I Efanov J , Arsenault J , et al. The partnership co‐design lab: co‐constructing a patient advisor programme to increase adherence to rehabilitation after upper extremity replantation. J Health Des. 2018;3(1):94‐101. https://www.journalofhealthdesign.com/JHD/article/view/47

[15] Pomey MP , de Guise M , Desforges M , et al. The patient advisor, an organizational resource as a lever for an enhanced oncology patient experience (PAROLE‐onco): a longitudinal multiple case study protocol. BMC Health Serv Res. 2021;21(10):10. 10.1186/s12913-020-06009-4 33397386 PMC7780212

[16] Braun V , Clarke V . Thematic analysis. In: Cooper H , Camic PM , Long DL , Panter AT , Rindskopf D , Sher KJ , eds. APA Handbook of Research Methods in Psychology, Vol 2: Research Designs: Quantitative, Qualitative, Neuropsychological, and Biological. APA handbooks in psychology®. American Psychological Association; 2012:57‐71. 10.1037/13620-004

[17] Kiger ME , Varpio L . Thematic analysis of qualitative data: AMEE Guide No. 131. Med Teach. 2020;42(8):846‐854. 10.1080/0142159X.2020.1755030 32356468

[18] Dunn J , Steginga SK , Rosoman N , Millichap D . A review of peer support in the context of cancer. J Psychosoc Oncol. 2003;21(2):55‐67. 10.1300/J077v21n02_04

[19] Brodar KE , Carlisle V , Tang PY , Fisher EB . Identification and characterization of peer support for cancer prevention and care: a practice review. J Cancer Educ. 2022;37(3):645‐654. 10.1007/s13187-020-01861-8 32892278 PMC7474572

[20] Kowitt SD , Ellis KR , Carlisle V , et al. Peer support opportunities across the cancer care continuum: a systematic scoping review of recent peer‐reviewed literature. Support Care Cancer. 2019;27(1):97‐108. 10.1007/s00520-018-4479-4 30293093 PMC6561346

[21] Hu J , Wang X , Guo S , et al. Peer support interventions for breast cancer patients: a systematic review. Breast Cancer Res Treat. 2019;174(2):325‐341. 10.1007/s10549-018-5033-2 30600413

[22] Fisher EB , Ballesteros J , Bhushan N , et al. Key features of peer support in chronic disease prevention and management. Health Aff. 2015;34(9):1523‐1530. 10.1377/hlthaff.2015.0365 26355054

[23] Jacobson N , Trojanowski L , Dewa CS . What do peer support workers do? A job description. BMC Health Serv Res. 2012;12(1):205. 10.1186/1472-6963-12-205 22812608 PMC3483205

[24] Allicock M , Carr C , Johnson LS , et al. Implementing a one‐on‐one peer support program for cancer survivors using a motivational interviewing approach: results and lessons learned. J Cancer Educ. 2014;29(1):91‐98. 10.1007/s13187-013-0552-3 24078346 PMC4066630

[25] Mirrielees JA , Breckheimer KR , White TA , et al. Breast cancer survivor advocacy at a university hospital: development of a peer support program with evaluation by patients, advocates and clinicians. J Cancer Educ. 2017;32(1):97‐104. 10.1007/s13187-015-0932-y 26477478 PMC4837082

[26] Braun KL , Kagawa‐Singer M , Holden AEC , et al. Cancer patient navigator tasks across the cancer care continuum. J Health Care Poor Underserved. 2012;23(1):398‐413. 10.1353/hpu.2012.0029 22423178 PMC3302357

[27] Peart A , Lewis V , Brown T , Russell G . Patient navigators facilitating access to primary care: a scoping review. BMJ Open. 2018;8(3):e019252. 10.1136/bmjopen-2017-019252 PMC5875656 29550777

[28] Phillips S , Villalobos AVK , Crawbuck GSN , Pratt‐Chapman ML . In their own words: patient navigator roles in culturally sensitive cancer care. Support Care Cancer. 2019;27(5):1655‐1662. 10.1007/s00520-018-4407-7 30109486 PMC6449285

[29] Gillard S , Foster R , Gibson S , Goldsmith L , Marks J , White S . Describing a principles‐based approach to developing and evaluating peer worker roles as peer support moves into mainstream mental health services. Ment Health Soc Incl. 2017;21(3):133‐143. 10.1108/MHSI-03-2017-0016

[30] Jacobson T , Mackey T . Proposing a metaliteracy model to redefine information literacy. Commun Inf Technol. 2013;7(2):84. 10.15760/comminfolit.2013.7.2.138

[31] Huntingdon B , Schofield P , Wolfowicz Z , et al. Toward structured peer support interventions in oncology: a qualitative insight into the experiences of gynaecological cancer survivors providing peer support. Support Care Cancer. 2016;24(2):849‐856 26223321 10.1007/s00520-015-2853-z

[32] Pomey MP , Ghadiri DP , Karazivan P , Fernandez N , Clavel N . Patients as partners: a qualitative study of patients' engagement in their health care. PLoS One. 2015;10(4):e0122499. 10.1371/journal.pone.0122499 25856569 PMC4391791

[33] Pomey MP , Clavel N , Normandin L , et al. Assessing and promoting partnership between patients and health‐care professionals: co‐construction of the CADICEE tool for patients and their relatives. Health Expect. 2021;24(4):1230‐1241. 10.1111/hex.13253 33949739 PMC8369086

[34] Solomon P . Peer support/peer provided services underlying processes, benefits, and critical ingredients. Psychiatr Rehabil J. 2004;27(4):392‐401. 10.2975/27.2004.392.401 15222150

[35] Gates LB , Akabas SH . Developing strategies to integrate peer providers into the staff of mental health agencies. Adm Policy Ment Health. 2007;34(3):293‐306. 10.1007/s10488-006-0109-4 17340184

