# Supplementary material for: Accompanying patients in clinical oncology teams: Reported activities and perceived effects
Source: Health Expect. 2023 Jan 26;26(2):847–57. doi: 10.1111/hex.13710 (PMC10010089; doi:10.1111/hex.13710)
Supplement: Supplementary file 1 — Supporting information. [file HEX-26--s001.docx]

Guide d’entrevue PA

Début de projet - 2019

(***Exploration complémentaire – 2020***)

PRÉAMBULE

**Formulaire d'information et de consentement**

- S’assurer que le participant présent a bien signé le FIC.
- S’assurer que le participant accepte que l’entrevue soit enregistrée.

**[Débuter l’enregistrement]**

Ce projet de recherche vise à évaluer l’implantation de patients accompagnateurs en oncologie dans les équipes de soins. Vous avez l’occasion de vous exprimer et de partager vos expériences et opinions sur le sujet au cours de cette rencontre. Nous fonctionnerons à partir de certaines questions, que je vous présenterai tantôt. Les thématiques qui seront abordées sont les suivantes : définition du PA, facteurs influençant l’intervention, les effets et les enjeux.

**Règles**

- Durée maximale de 45 minutes.
- Annonce des enregistrements audio et vidéo
- Explication de la présence des observateurs à distance et raisons
- Fermeture des cellulaires
- Respect de l’anonymat et de la confidentialité et identification des participantes par leur prénom seulement
- Pas de bonnes ou de mauvaises réponses

QUESTIONS

1. Comment voyez-vous votre intégration dans l’équipe de soins ?

*Pour le modérateur : amener à définir clairement comment le PA va être intégré ou est intégré.*

1. *Selon vous, quel est le rôle de la patiente accompagnatrice ?*

*Qu’est-ce qui vous distingue des autres membres de l’équipe de soins ?*

*Selon vous, comment les membres de l’équipe de soins vous perçoivent-ils ?*

*Comment décririez-vous la relation que vous avez développée avec les différents membres de l’équipe clinique ?*

*Selon vous, avec quel type de professionnels de l’équipe de soins est-il plus facile de développer un lien ou une relation ? Pour quelles raisons ?*

1. Selon vous, quels **facteurs** pourraient favoriser l’intégration **des PA** ?
2. Selon vous, quels **facteurs pourraient** nuire à l’intégration **des PA** ?

*Dans un monde idéal, quelles devraient être les ressources spécifiques (humaines, financières, d’infrastructure ou informationnelles) allouées par l’établissement pour l’intégration des PA ? Parler des enjeux juridiques et éthiques.*

1. Selon vous, quels sont/seront les **effets** de l’intégration **des PA** ?

*Pour le modérateur : Quelles sont les dimensions qui sont concernées selon vous ?*

- - *Sur les patients (Symptômes maladie/Qualité de vie, Observance, Expérience de soin, Partenariat et activation, Utilisation du système de santé)*
  - *Sur les PA (Donne du sens à leur histoire, Utilité sociale, Deuil)*
  - *Sur l’équipe (Partenariat de soins, Pratiques collaborative, Amélioration et transfert des connaissances, Intérêt à travailler avec des PA) ou l’organisation (Refonte des parcours de soins)*

1. Avez-vous d’autre chose que vous aimeriez ajouter ? D’autres sujets que vous aimeriez aborder et que vous jugez important d’explorer ?

*Merci beaucoup de votre participation!*
